# Oral challenge vs routine care to assess low-risk penicillin allergy in critically ill hospital patients (ORACLE): a pilot randomised controlled trial

**DOI:** 10.1186/s40814-023-01337-8

**Published:** 2023-07-20

**Authors:** Morgan Rose, Natasha Holmes, Glenn Eastwood, Sara Vogrin, Fiona James, Michelle Phung, Sara Barnes, Brendan Murfin, Ben Rogers, Belinda Lambros, Trisha Peel, Grace Gibney, Monica Slavin, Jason Trubiano

**Affiliations:** 1 Centre for Antibiotic Allergy and Research, Department of Infectious Diseases, Austin Health, Level 7, Harold Stokes Building, 145 Studley Road, Heidelberg, VIC 3084 Australia; 2 National Centre for Infections in Cancer, Peter MacCallum Cancer Centre, Melbourne, VIC Australia; 3 Department of Infectious Diseases, Peter MacCallum Cancer Centre, Melbourne, VIC Australia; 4 Department of Infectious Diseases, University of Melbourne, at the Peter Doherty Institute for Infection and Immunity, Melbourne, VIC Australia; 5 Data Analytics Research and Evaluation Centre, Austin Health/University of Melbourne, Heidelberg, VIC Australia; 6 Department of Critical Care, The University of Melbourne, Parkville, VIC Australia; 7 Department of Intensive Care, Austin Hospital, Heidelberg, VIC Australia; 8 Department of Medicine (St Vincent’s Hospital), University of Melbourne, Melbourne, VIC Australia; 9 Pharmacy Department, Monash Health, Clayton, VIC Australia; 10 Faculty of Medicine, Nursing and Health Sciences, Monash University, Clayton, VIC Australia; 11 Monash Lung Sleep Allergy and Immunology, Monash Health, Clayton, VIC Australia; 12 Intensive Care Unit, Monash Health, Clayton, VIC Australia; 13 Monash Infectious Diseases, Monash Health, Clayton, VIC Australia; 14 School of Clinical Sciences, Monash University, Clayton, VIC Australia; 15 Department of Infectious Diseases, Central Clinical School, Faculty of Medicine, Nursing and Health Sciences, Monash University, Melbourne, VIC Australia; 16 Department of Infectious Diseases, Alfred Health, Melbourne, VIC Australia; 17 Sir Peter MacCallum Department of Oncology, University of Melbourne, Parkville, VIC Australia; 18 Immunocompromised Host Infection Service, Victorian Infectious Diseases Service, Royal Melbourne Hospital, Melbourne, VIC Australia

**Keywords:** Allergy, Delabelling, Antibiotics, Penicillin, Intensive care, Critical illness

## Abstract

**Background:**

Self-reported penicillin allergies are highly prevalent in hospitalised patients and are associated with poor health and health service outcomes. Critically ill patients have historically been underrepresented in prospective delabelling studies in part due to concerns around clinical stability and reliability of penicillin skin testing. Allergy assessment tools exist to identify low-risk penicillin allergy phenotypes and facilitate direct oral challenge delabelling. PEN-FAST is a clinical decision rule that has been validated to predict true penicillin allergy in a cohort of non-critically ill patients. There is however limited evidence regarding the feasibility, safety and efficacy of direct oral challenges and the use of delabelling clinical decisions rules in the intensive care setting.

**Methods:**

Critically ill patients in the intensive care unit (ICU) with low-risk penicillin allergy phenotypes (PEN-FAST score < 3) will be randomised 1:1 to direct oral penicillin challenge (single dose 250 mg oral amoxicillin or implicated penicillin) or routine care, followed by a 2-h observation period. Patients will receive a second oral challenge/observation prior to hospital discharge (with subsequent observation for 2 h). An assessment for antibiotic-associated adverse events will also be undertaken at 24 h and 5 days post each challenge/observation and again at 90 days post-randomisation. The primary outcome measures are feasibility (proportion of eligible patients recruited and protocol compliance) and safety (proportion of patients who experience an antibiotic-associated immune-mediated adverse event or serious adverse event).

**Discussion:**

We will report the feasibility and safety of point-of-care penicillin direct oral challenge in this first randomised controlled trial of low-risk penicillin allergy in critically ill hospitalised patients. Upon completion of the project, important findings will inform the design of planned large prospective multi-centre clinical trials in Australian and international ICUs, further examining safety and efficacy and exploring antimicrobial prescribing-related outcomes following penicillin oral challenge.

**Trial registration:**

Australian New Zealand Clinical Trials Registry

Registration Number: ACTRN12621000051842

Date registered: 20/01/2021

https://www.anzctr.org.au/Trial/Registration/TrialReview.aspx?id=379735&isReview=true

**Supplementary Information:**

The online version contains supplementary material available at 10.1186/s40814-023-01337-8.

## Background

Patient-reported penicillin allergies result in poor health outcomes for patients and drive inappropriate antibiotic prescribing, antimicrobial resistance and healthcare costs [1–5]. Critically ill patients, such as those in the intensive care unit (ICU), are especially vulnerable to the impact of penicillin allergies [6, 7], yet have been underrepresented in interventional prospective delabelling (i.e. the removal of a patient’s antibiotic allergy history (“label”) via testing or medical record reconciliation [8]) programmes [9]. This impact is magnified, as the prevalence of penicillin allergies is highest in the critical care setting (9–15%) [1], with 50% of these considered low risk and amenable to point-of-care delabelling [3]. Despite the described burden of low-risk penicillin antibiotic allergies being highest in the ICU setting, these have not been addressed in controlled interventional studies.

We recently demonstrated in an Australian ICU that antibiotic allergies are associated with inferior prescribing, in particular the excessive utilisation of vancomycin (aOR 2.04; 95% CI 1.07, 3.86) and inadequate use of narrow spectrum beta-lactams (aOR 0.52; 95% CI 0.29, 0.94) [5]. In a range of observational studies that have included ICU patients, penicillin allergies are associated with the increased use of restricted antibiotics [5].

Our previous work has shown that more than 85% of penicillin allergies can be removed by formal skin prick allergy testing [10], and 96–98% with low-risk allergies can be removed by point-of-care oral challenge (i.e. test dose in non-ICU hospitalised patients) [11, 12]. However, the safety and efficacy of oral challenge in critical care are ill-defined [5]. In a recent systematic review, Moran et al. identified that penicillin allergy testing in the ICU was associated with increased use of narrow-spectrum antibiotics and decreased utilisation of restricted antimicrobials [5]. In addition, in a single-centre study, narrow-spectrum beta-lactams were utilised in 39.5% of ICU patients reporting any antibiotic allergy compared with 58.8% without (*p* = 0.028) [6].

Traditional penicillin allergy skin testing in the ICU has challenges, including the potential for false negatives, resourcing requirements and time pressures [5].

As an alternative to traditional assessment, we have validated the use of an antibiotic allergy assessment tool and point-of-care direct oral challenge in non-critically ill hospitalised inpatients (*n* = 196, 98.9% efficacy) [12]. Further, a recent unmatched single-centre cohort study demonstrated the safe administration of single-dose direct amoxicillin challenge in ICU patients with low-risk penicillin allergy (*n* = 54, 100% uncomplicated) [13]. Finally, our group has internally and externally validated a novel penicillin allergy clinician decision rule (PEN-FAST) that is able to identify low-risk penicillin allergies with a negative predictive value (NPV) of 96% (95% CI 94–98%) for a score of < 3 [14]. Therefore, whilst validated tools exist to enable inpatient penicillin assessment and delabelling, limited evidence is available regarding the safety and efficacy in the ICU setting.

## Methods/design

### Study objectives

To investigate the feasibility and safety of a direct oral penicillin challenge in adult ICU patients that have a low-risk penicillin allergy (PEN-FAST score < 3). Outcome measures are listed in Table 1 and include the primary outcomes of safety and feasibility of direct oral challenge. Secondary outcomes include proportion of allergy labels removed post-oral challenge, proportion of positive repeat oral challenges, pre- and post-randomisation antimicrobial utilisation, in-hospital and 30-day mortality and length of stay.

**Table 1:** Outcome measures

**Primary outcome measures**	
* Feasibility outcome measures*	
* Eligibility to screened ratio*	Proportion of patients that are eligible for intervention
*** Recruitment to eligibility ratio***	Proportion of eligible patients consenting to the participation in the study *(a ratio of* ≥ *50% will be used as the primary determinant of feasibility)*
* Intervention to recruitment ratio*	Proportion of patients randomised to the intervention arm that had the intervention delivered as per protocol
* Protocol compliance*	Proportion of randomised patients that complete all study activities as per protocol
* Safety outcome measures*	
* Safety*	Proportion of patients with a penicillin allergy who experience an antibiotic-associated immune-mediated adverse event OR serious adverse event *(a proportion of* < *5% will be used as the determinant of safety)*
**Exploratory outcome measures**	
◦ *Proportion of participants in the intervention arm successfully delabelled post oral challenge* ◦ *Proportion of participants in the intervention arm with positive repeat oral challenge (post-ICU discharge)* ◦ *Utilisation of any penicillin during hospital admission* ◦ *Utilisation of any narrow-spectrum beta-lactam during hospital admission* ◦ *Utilisation of vancomycin during hospital admission* ◦ *Utilisation of any restricted antibiotic during hospital admission* ◦ *In-Hhspital and 30-day mortality* ◦ *ICU length of stay and hospital length of stay*	

### Design and schedule

**Fig. 1:**
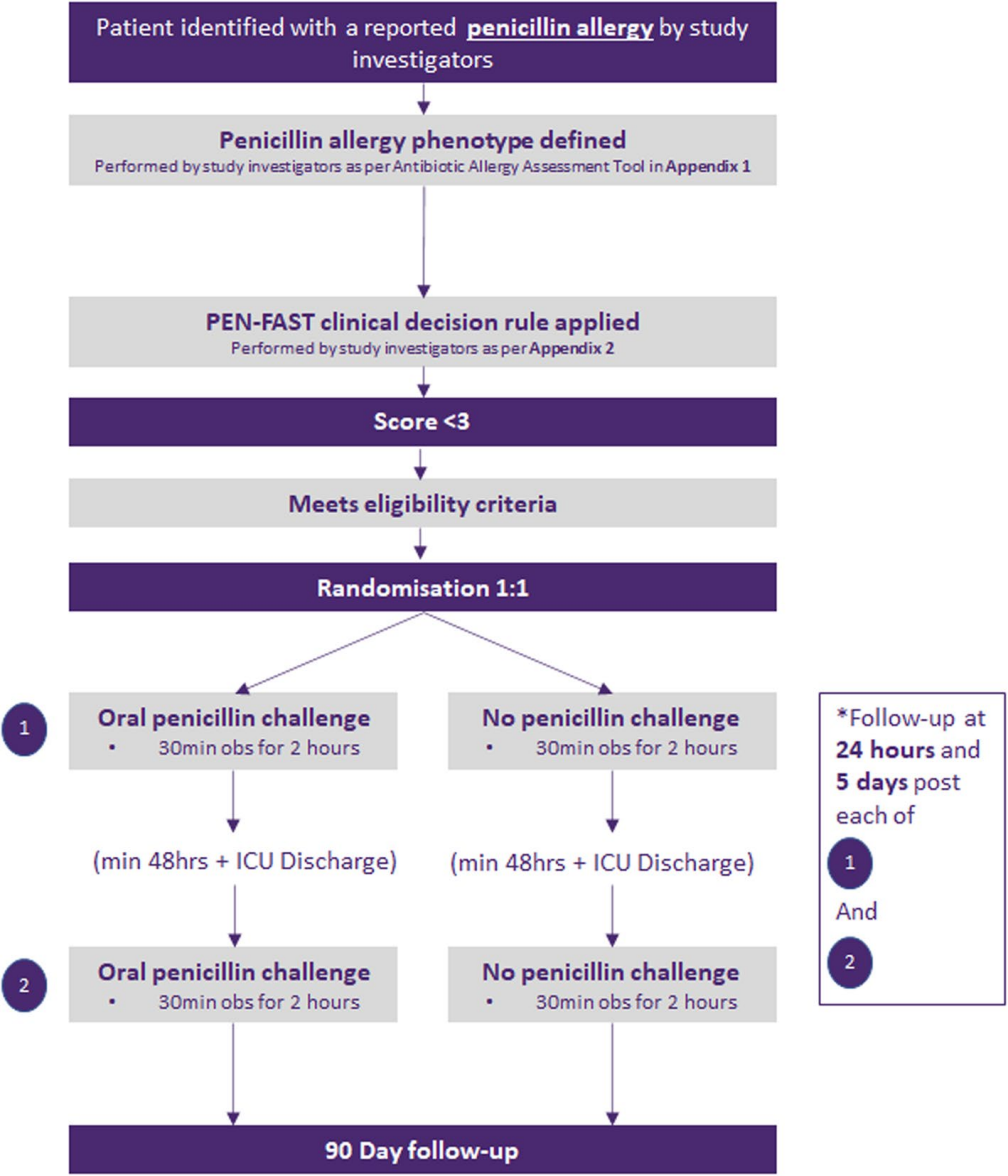
Summary of ORACLE study design

This is a pilot, feasibility, open-label randomised clinical trial that will be conducted in mixed medical/surgical ICUs in five tertiary referral teaching hospitals in Melbourne, Australia (Austin Health, Peter MacCallum Cancer Centre, Melbourne Health, Monash Health, Alfred Health). A summary of the study design is presented in Fig. 1. We will include 80 patients with a low-risk penicillin allergy and allocate them in a 1:1 ratio to the intervention group (oral penicillin challenge) and control group (standard of care). Oral challenge will be undertaken using the implicated oral penicillin if known (including penicillin VK, amoxicillin, flucloxacillin, dicloxacillin). Challenge for “penicillin unspecified” allergy labels will utilise amoxicillin (the most common community-prescribed oral penicillin in Australia) [15] or penicillin VK (if the reaction occurred prior to amoxicillin availability).

Adult patients admitted to the ICU reporting a penicillin allergy will be identified by study investigators from a daily electronic medical record (EMR) list and assessed utilising a validated Antibiotic Allergy Assessment Tool (Additional file 1) to obtain allergy phenotype [16]. Once the reported allergy phenotype has been determined, the PEN-FAST clinical decision rule will be applied (Additional file 2). Informed consent will be sought from those with a PEN-FAST score < 3 who meet all eligibility criteria, and they will be randomised to the intervention arm or control arm. All other concomitant care and interventions will be undertaken according to local ICU and hospital protocols.

### Eligibility criteria

#### Inclusion criteria

Inclusion criteria will include adult (≥ 18 years) ICU inpatients reporting a penicillin allergy with a PEN-FAST assessment score of less than 3 (representing a low or very low risk of true penicillin allergy) and an expected ICU discharge date of > 24 h post-randomisation.

#### Exclusion criteria

Patients will be excluded if their allergy history cannot be confirmed, they are severely critically ill (unlikely to survive index admission, high ventilatory support requirements, high inotropic support requirements), with confirmed pregnancy, they are receiving medication that may interfere with allergy assessment (antihistamines, high-dose steroids), or they report a history of non-penicillin drug-associated anaphylaxis or idiopathic urticaria/anaphylaxis/mastocytosis.

A full list of inclusion and exclusion criteria is presented in Table 2.

**Table 2:** ORACLE study eligibility criteria

**Inclusion criteria**
1. Adult (≥ 18 years) ICU inpatient
2. Reported penicillin allergy with a PEN-FAST score < 3
3. Are expected to stay in the ICU at least 24 h post-assessment
**Exclusion criteria** (patient excluded if **ONE** of the following criteria present)
1. Known pregnancy
2. Death is deemed imminent or inevitable during this admission, and either the attending physician, patient or medical treatment decision-maker is not committed to active treatment
3. Any other illness that, in the investigator’s judgement, will substantially increase the risk associated with subject’s participation in this study
4. Patients with known history of ANY non-penicillin drug-associated anaphylaxis
5. Patients with a known history of idiopathic urticaria, idiopathic anaphylaxis or mastocytosis
6. Patients where the allergy history was not able to be confirmed with patient or medical treatment decision-maker
7. Patients on antihistamine therapy (excluding H2-receptor antagonists)
8. Patients receiving > 10 µg/minute of noradrenaline or any adrenaline therapy in the last 4 h
9. High ventilator requirement if intubated (any of the following) i. Any mode other than spontaneous ii. Peak end-expiratory pressure (PEEP) > 5 cm H2O iii. FiO2 > 40%
10. Patients receiving more than stress dose steroid therapy (i.e. > 50 mg QID hydrocortisone or daily equivalent)

### Intervention and control arms

#### Intervention

The intervention is a single dose 250 mg of oral penicillin (capsule or liquid via enteral route, including nasogastric/PEG/PEJ) following baseline observations being performed (i.e. temperature, heart rate, blood pressure, respiratory rate, skin check). The implicated penicillin will be administered if known. Amoxicillin will be administered for “penicillin unspecified” allergy labels (or penicillin VK if the reaction occurred prior to the availability of amoxicillin). The penicillin challenge dose will be charted by the study or ICU clinician (following review of baseline observations) and administered by bedside nursing staff. Clinical observations will be collected by the bedside nursing staff at + 30, + 60, + 90 and + 120 min post-oral challenge. If at any stage an antibiotic-associated adverse event is noted, the treating and study clinicians will be informed. Treatment of the adverse event will be at the discretion of the treating clinician.

Patients will undergo a repeat single-dose oral penicillin (250 mg) challenge (if initial challenge is negative) following ICU discharge and at least 48 h post-initial challenge (if they remain an inpatient). Participants who no longer require ICU-level care but have not yet been transferred out of the unit due to bed availability may receive the repeat oral challenge within the ICU if at least 48 h have passed since the primary challenge dose. A further set of clinical observations at 30-min intervals over 2 h will be collected following the repeat oral challenge. The repeat challenge is administered to assess for a potential false-negative initial oral challenge secondary to critical illness. Participants will be reviewed at 24 h and 5 days post-randomisation; 24 h and 5 days post the second oral challenge and 90 days post-randomisation. Participant review will assess for any serious or antibiotic-associated adverse events as per protocol definitions and will be undertaken in-person whilst an inpatient or via telephone or telehealth following hospital discharge. Participants with a positive initial challenge will not proceed to a repeat challenge.

#### Control

Routine management of penicillin allergy label as per local ICU protocols without oral penicillin challenge. Patients in the control arm will have observations performed at randomisation and at + 30, + 60, + 90 and + 120 min post-randomisation by the bedside nursing staff. A further set of clinical observations at 30-min intervals over 2 h will be collected following ICU discharge and at least 48 h after randomisation (if they remain an inpatient). Participants will be reviewed at 24 h and 5 days post-randomisation, 24 h and 5 days post the second observation period and 90 days post-randomisation. Participant review will assess for any serious or antibiotic-associated adverse events as per protocol definitions and will be undertaken in-person whilst an inpatient or via telephone or telehealth following hospital discharge. After the completion of the 90-day review, all participants in the control arm will be offered a referral to their nearest public antibiotic allergy assessment clinic for definitive assessment of their penicillin allergy label.

### Participant timeline

**Fig. 2:**
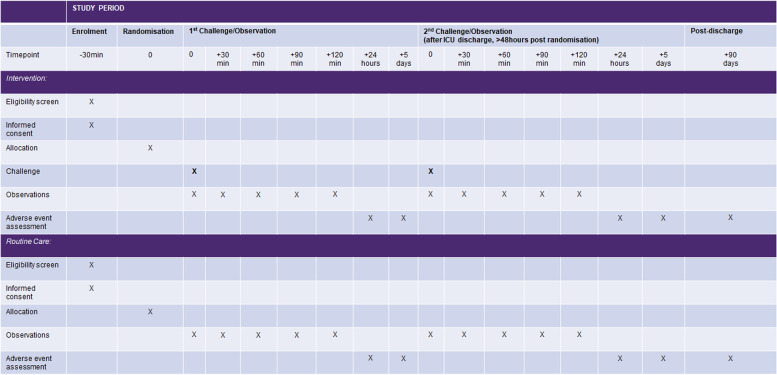
ORACLE study participant timeline

A timeline of the enrolment, interventions and review of participants in the intervention and control arms is presented in Fig. 2.

### Sample size

This is a pilot study designed to assess the feasibility and safety of the ICU oral challenge programme, as well as providing local estimates for subsequent power calculation for future trials.

With regard to the feasibility of penicillin allergy assessment within the ICU, we assume that 9% of all screened patients will report a penicillin allergy as per national data [1], and in an observational study by Moran et al. in Austin Health ICU [6], 200 patients would be expected to be eligible in a 12-month study period. Assuming 50% recruitment and that 85% complete the randomised challenge/observation, this would generate a projected potential pool of 85 eligible participants in 1 year. As such, a total of 80 enrolments and 1:1 randomisation (40 in each arm) are planned.

Whilst the above assumptions may be true (particularly for the primary study site, with an established clinical and research antibiotic allergy service), there are anticipated factors which may limit recruitment. The expected number of 200 eligible patients was calculated from a single tertiary ICU, and the different patient populations of other ICUs may vary the number of eligible patients. Temporary disruptions are expected due to the ongoing SARSCo-V2 pandemic, with altered patient flow through ICUs and investigator availability. Delays in activating study sites may slow recruitment and consistent recruitment may be more challenging at sites with less robust allergy research services.

### Randomisation

Randomisation will be determined by means of an electronically generated allocation sequence using permuted block design, stratified by clinical site. Allocation concealment will be achieved via opaque sealed sequentially numbered envelopes at a single study site (Austin Health). All other study sites will undertake allocation using REDCap (Research Electronic Data Capture).

At Austin Health, generation of the randomisation sequence for the sequentially numbered envelopes will be performed by independent ICU research coordination staff.

The REDCap allocation sequence will be developed and uploaded to REDCap by a trial statistician and will remain concealed prior to allocation.

Allocation will be performed by study investigators (unsealing the opaque envelopes at Austin Health or via the REDCap database randomisation tool at all other sites).

### Blinding

The participant, ICU team and treating clinicians will be blinded to the formal Allergy Assessment Tool result and PEN-FAST score. The participant, ICU team and treating clinicians will not, however, be blinded to the result of eligibility to be randomised (i.e. low risk vs. high risk). The participant, ICU team and treating clinicians will not be blinded to the allocation or outcome of the oral penicillin challenge in participants of the intervention arm.

### Data collection

For all participants, data will be collected during the study by investigators as per the electronic Case Record Form (REDCap). Data to be collected will include basic demographics, medical history, details of the index hospital admission (including diagnosis, critical illness severity, infective episodes and antimicrobial treatment and hospital and ICU length of stay), antimicrobial allergy history, penicillin challenge and/or observation data and any identified antibiotic-associated adverse events during the study period (including serious adverse events). A full list of data points is listed in Additional file 3.

### Statistical methods

Data analysis will be performed on an intention-to-treat basis as well as per protocol. Summary statistics will be used to describe the clinical data and presented as mean ± standard deviation (SD), median with interquartile range (IQR) or percentages as appropriate.

Feasibility and safety outcomes will be reported as percentage with 95% confidence intervals. Logistic regression will be used to compare antibiotic utilisation and mortality between groups. Results will be reported as odds ratios with 95% confidence intervals. Negative binomial regression will be used for comparison of length of stay (reported as incidence rate ratio with 95% CI). Results will be reported according to CONSORT guidelines [17].

#### Data monitoring

As a pilot study with a small number of planned participants utilising an established safe allergy assessment protocol, a data monitoring committee will not be established. Any serious adverse events or antibiotic-associated immune-mediated adverse events will be referred to two independent clinicians with extensive experience in antibiotic allergy for adjudication.

Participant retention will be promoted through the offer of telephone follow-up for patients post-hospital discharge.

#### Harms

Serious adverse events (SAEs) will be defined as any adverse drug event that, in the opinion of the investigators, is causal for any of these outcomes: (1) death; (2) life-threatening reaction; (3) inpatient hospitalisation; (4) results in persistent or significant disability/incapacity; (5) congenital anomaly or birth defect; or (6) requires intervention to prevent permanent impairment or damage. SAEs that occur from the time of commencement of study treatment to 5 days after the second challenge/observation will be collected and reported to the approving ethics committee within 24 h of study staff becoming aware of the event.

An antibiotic-associated immune-mediated adverse event will include any immune-mediated reaction within 48 h of an antibiotic dose judged by two independent reviewers. An antibiotic-associated adverse event will be defined as any non-immune mediated reaction (e.g. diarrhoea, nausea and vomiting) within 48 h of an antibiotic dose judged by two independent reviewers.

### Confidentiality and dissemination

#### Confidentiality

Paper data and study-related documents used in this study will be de-identified and only a master log will be maintained to identify participants and their study data. The log will be locked in a secure office. Electronic data will be stored in a password-protected REDCap database hosted by Austin Health. All data for study will be retained for a period of 15 years after which all electronic and paper data will be destroyed in accordance with hospital policy in place at the time. Only aggregated non-identifiable patient data will be presented or published.

#### Dissemination policy

The results of this pilot study will be submitted for publication in peer-reviewed journals.

## Discussion

Penicillin allergy labels are very common in the critical care setting and are associated with significant negative patient and healthcare outcomes [5, 6]. Existing antibiotic allergy assessment and testing programmes have been shown to effectively remove penicillin allergy labels [12]. Point-of-care testing within the ICU has the potential to assess penicillin allergies in a closely monitored environment and reduce the negative effects of allergy labels by promoting β-lactam antimicrobials and limiting exposure to non-preferred alternative antibiotics. However, critically ill patients have been underrepresented in delabelling programmes to date. Furthermore, traditional methods of in vivo allergy assessment (skin testing) have been challenging to implement in critical care patients, with concerns being raised regarding false-negative tests and increased rates of negative histamine control results [9]. This has led some allergy services to focus on the assessment of low-risk penicillin allergy labels in critical illness through direct oral challenge programmes, where skin testing is not required.

Early uncontrolled cohort data provides support for the safety and efficacy of direct oral penicillin challenge in critical illness [13]. However, this study represents the first randomised controlled trial to assess the feasibility, safety and efficacy of a validated point-of-care allergy assessment tool and oral challenge programme in critical illness. Further, through administering repeat challenges post-ICU discharge, this study will provide robust early data on the impact of critical illness on the validity of direct oral challenge. Finally, exploratory data on the impact of oral challenge on antimicrobial prescribing will be collected through detailed electronic medical review and patient telephone review in both the oral challenge and routine care cohorts.

This study will provide feasibility, initial safety and exploratory efficacy data for direct oral challenge for low-risk penicillin allergy in ICU. The findings of this study will be translated into future large-scale, multi-site randomised studies in Australian and international ICUs to more fully explore the impact of oral challenge in ICU on antimicrobial prescribing and patient and health service outcomes and the optimal model for antibiotic allergy assessment in the critical care setting.

## Supplementary Information

**Additional file 1.** Antibiotic Allergy Assessment Tool.

**Additional file 2.** PEN-FAST.

**Additional file 3.** Data fields per participant.

## Data Availability

Data sharing is not applicable to this article as no datasets were generated or analysed during the current study.
