# Supplementary material for: Oral challenge vs routine care to assess low-risk penicillin allergy in critically ill hospital patients (ORACLE): a pilot randomised controlled trial
Source: Pilot Feasibility Stud. 2023 Jul 20;9:126. doi: 10.1186/s40814-023-01337-8 (PMC10357614; doi:10.1186/s40814-023-01337-8)

# **Additional file 1 – Antibiotic Allergy Assessment Tool**

**Penicillin allergy assessment questions**

1. What is the name of the oral penicillin?

Penicillin unspecified □ Penicillin VK **□** Penicillin G **□** Amoxicillin **□**

Ampicillin □ Dicloxacillin **□** Flucloxacillin **□** Nafcillin **□**

Oxacillin □ Benzathine **□** Amoxicillin clavulanate **□**

1. Please describe the details of this reaction. (*See descriptions in tool)*
2. How many years ago did the reaction occur?

More than 5 years ago? Yes **□** No **□**

More than 10 years ago? Yes **□** No **□**

1. How long after having the first antibiotic dose did the reaction occur?

Immediate (within 1-2 hours)? Yes **□** No **□**

1. Did it require any systemic treatment?

Yes **□** No **□**

1. Were you hospitalised as a result of this reaction? Yes **□** No **□**


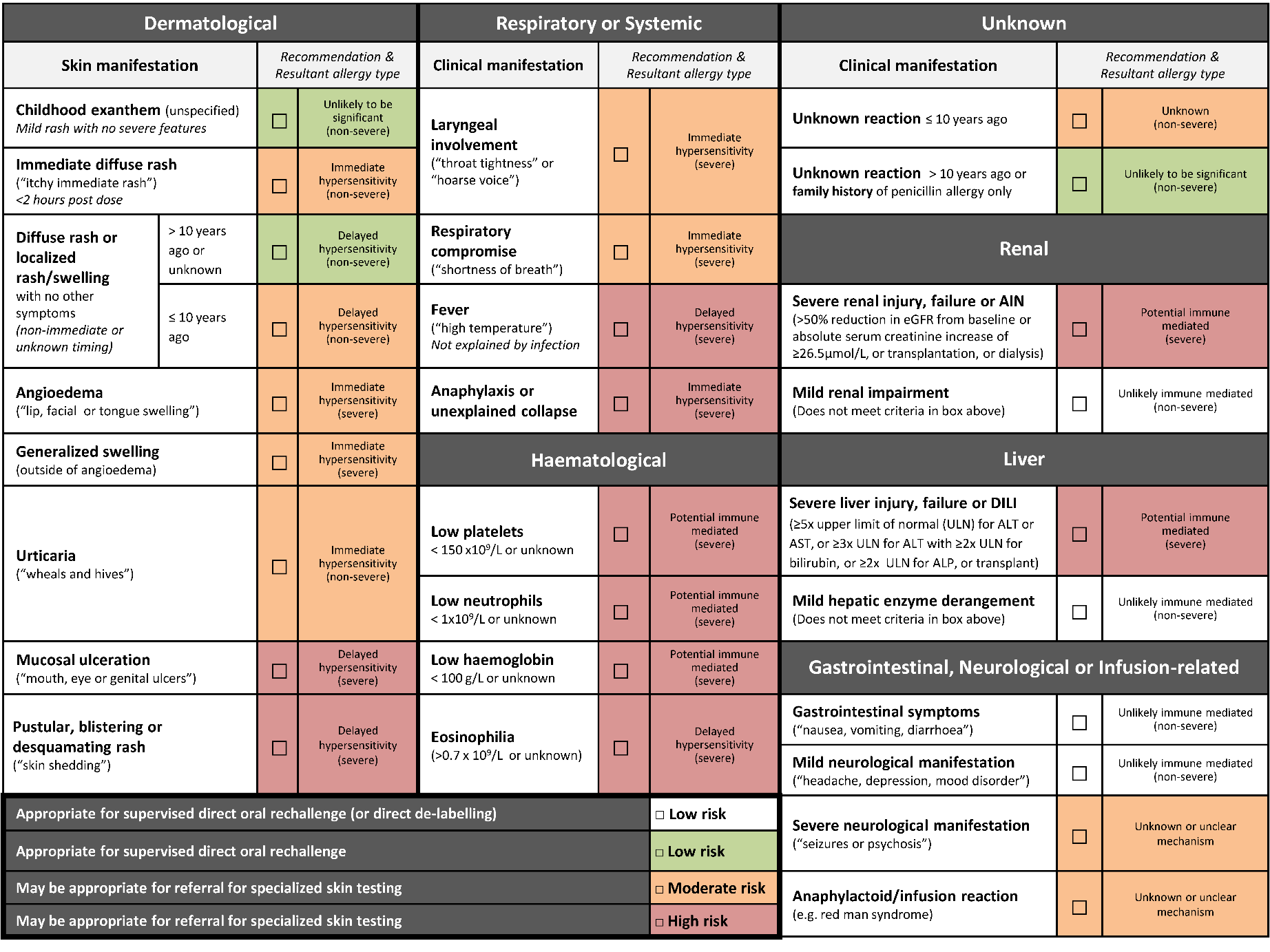

Supplement: Supplementary file 1 — Additional file 1. Antibiotic Allergy Assessment Tool. [file 40814_2023_1337_MOESM1_ESM.docx]
