# Supplementary material for: Oral challenge vs routine care to assess low-risk penicillin allergy in critically ill hospital patients (ORACLE): a pilot randomised controlled trial
Source: Pilot Feasibility Stud. 2023 Jul 20;9:126. doi: 10.1186/s40814-023-01337-8 (PMC10357614; doi:10.1186/s40814-023-01337-8)
Supplement: Supplementary file 2 — Additional file 2. PEN-FAST. [file 40814_2023_1337_MOESM2_ESM.docx]

# **Additional file 2 – PEN-FAST**

*
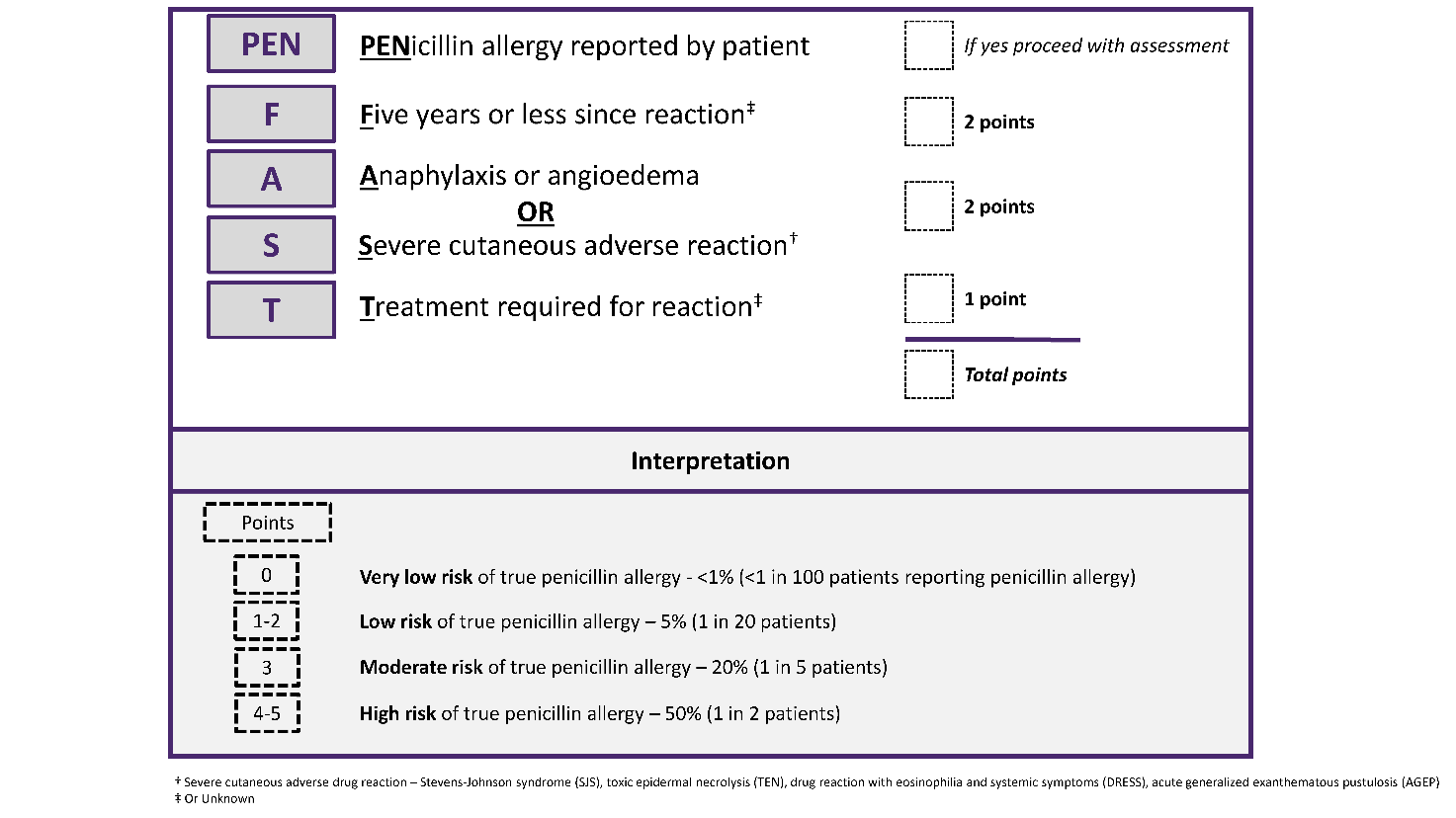
*

† Stevens-Johnson Syndrome, Toxic Epidermal Necrolysis, Drug Reaction with Systemic Symptoms, Acute Generalized Exanthematous or blistering or desquamating rash potentially consistent with SCAR.

‡ or unknown
