# Supplementary material for: Oral challenge vs routine care to assess low-risk penicillin allergy in critically ill hospital patients (ORACLE): a pilot randomised controlled trial
Source: Pilot Feasibility Stud. 2023 Jul 20;9:126. doi: 10.1186/s40814-023-01337-8 (PMC10357614; doi:10.1186/s40814-023-01337-8)
Supplement: Supplementary file 3 — Additional file 3. Data fields per participant. [file 40814_2023_1337_MOESM3_ESM.docx]

# **Additional file 3 – Data fields per participant**

| **Data Category** | **Data Points** |
| --- | --- |
| Baseline Demographics | Age |
|  | Date of birth |
|  | Sex |
|  | Ethnicity |
| Medical History | Age adjusted charlson comorbidity index (CCI) – immunosuppression history (including prolonged steroid therapy: defined as being on prednisolone or equivalent of >10mg/day for 1 month), cancer, haematological malignancy, autoimmune/rheumatological disorder requiring immunosuppression, transplant recipient, immune-uncontrolled HIV (defined as CD4+<200), chronic co-morbidities. |
| Admission Details | Hospital admission date |
|  | Hospital discharge date |
|  | Admitting unit |
|  | Pre-ICU ward/location |
|  | ICU admission date |
|  | ICU discharge date |
|  | Admission diagnosis |
|  | ICU admission diagnosis |
|  | APACHE II score (at ICU admission) |
|  | SOFA score (at ICU admission) |
|  | Infective episodes and antibiotic utilisation during index admission and post-discharge until day 90 post-randomisation (antibiotics: dose, frequency, duration, route, date/time of administration, appropriateness) |
|  | Mortality (in-hospital) |
|  | Mortality (30-day) |
| Allergy history | Listed antimicrobial(s) in allergy section of electronic medical record (EMR) |
|  | Listed allergy description in EMR |
|  | Any antibiotic allergy testing performed during study period |
|  | Antibiotic allergy phenotype (risk and type) as per Antibiotic allergy assessment tool (**Appendix 1.**) |
|  | PEN-FAST parameters and score (**Appendix 2.**) |
|  | Known tolerated antimicrobial(s) |
| Oral Challenge/Control Observations | Date of oral challenge/observation |
|  | The following data will be collected immediately prior to oral challenge/observation and then at 30-, 60-, 90- and 120-minutes post challenge/observation: Time, heart rate, blood pressure, mean arterial pressure, respiratory rate, oxygen saturation, FiO2, peak end expiratory pressure (if intubated), pressure support (if intubated) |
|  | Intervention arm only: antibiotic name, dose, time of administration, route of administration, outcome of oral challenge |
| Adverse drug reactions | Immune mediated [immediate (IgE) or non-immediate (T-cell)] reaction until day 90 post-randomisation |
|  | Serious adverse event(s) causally related to study intervention until day 5 post-2^nd^ challenge/observation |
